# Injection Molded PP Foams Using Food Ingredients for Food Packaging Applications

**DOI:** 10.3390/polym13020288

**Published:** 2021-01-18

**Authors:** Artemis Tsagdi, Ioannis Drossos, Despoina Georgiou, Stylianos Exarhopoulos, Georgios Karasiotas, Joannis K. Kallitsis, Eleni P. Kalogianni

**Affiliations:** 1 Department of Chemistry, University of Patras, GR-26504 Patras, Greece; artemis.tsagdh@gmail.com (A.T.); kallitsi@upatras.gr (J.K.K.); 2 Thrace Plastics Pack S.A., Member of Thrace Group Companies, GR-67100 Magiko Xanthi, Greece; ydrossos@thraceplastics.gr (I.D.); gkarasiotas@thraceplastics.gr (G.K.); 3 Department of Food Science and Technology, International Hellenic University, GR-57001 Thermi, Greece; dgeorgio@food.teithe.gr (D.G.); stelexar@food.teithe.gr (S.E.)

**Keywords:** polypropylene, coating process, foamed PP, foaming agents, food ingredients

## Abstract

A new approach to the creation of polypropylene (PP) based foaming materials was developed using food grade foaming agents that were coated on the PP pellets. More specifically, sodium bicarbonate and organic acids were used to coat PP pellets using either polyethyleneoxide (PEO) or lipid esters as coating stabilizers. In order to overcome the problem of the thermal decomposition of sodium bicarbonate at temperatures lower than the PP melting temperature, which makes the direct foaming during melt mixing impossible, the proposed methodology was proved quite efficient. Thus, new PP masterbatches were prepared, where the foaming agents were incorporated as coating at PP pellets at contents up to 10%, and initially used in Lab scale injection machines in order to find the best combination of materials that resulted in the production of foamed articles. Subsequently selected material combinations were tested in an industrial scale injection molding machine, where an optimization of the injection parameters was attempted. The outcome of this was the production of PP articles with significantly increased void fraction, up to 14%, decreased thermal conductivity, up to 20%, and various pore sizes as was observed via microscopic examination using SEM and CLSM.

## 1. Introduction

Foaming in thermoplastic polymers has attracted attention, since it provides the produced materials with a combination of desirable properties like thermal insulation and high specific strength, with low density [1]. Different foaming approaches like mechanical physical and chemical have been used [2,3,4]. The polymeric foams are also known as porous polymeric material, as they consist of a polymeric matrix, which contains an important number of small pores. Polymer foams can develop a different geometry, such as open cells or closed cells, depending on the raw materials and the method used for their creation. Polymeric foams—because of their advantages—can fit in different fields, such as packaging, thermal insulation systems, sporting equipment and automotive parts [5,6]. Polymeric foams find applications also in food packaging, providing lightweight insulating properties to the package [7]. The most commonly used polymeric foam used for food packaging is expanded polystyrene (EPS). EPS however poses environmental concerns since it is not degradable and is difficult to recycle [8].

Polyolefins are among the polymers used widely for food packaging applications and are considered as promising polymers for foaming because of their low cost and their recycling ability, as they are used in a variety of industrial applications. Polypropylene (PP) is one of the most popular thermoplastic materials. However, PP due to its linear structure and low melt strength shows difficulties in the foaming processing. For this reason, foaming of polypropylene is very challenging and a combination of physical and chemical foaming agents are required in some cases [6]. Furthermore, in cases that applications in food packaging are targeted, only foaming agents that are approved for foods can be used (e.g., EC 1935/2004 [9]). Such materials are inorganic compounds, such as sodium bicarbonate and their combination with organic acids which havedecomposition temperatures that are compatible with the polypropylene processing temperature [10,11,12,13,14]. Chemical foaming is an important foaming method and it is important to choose the range of the decomposition temperature of the foaming agent to match the processing temperature of the matrix polymer. The decomposition products of chemical blowing agents, which can be used in different temperature ranges, are various gases such as CO_2_ or N_2_. For material designed for contact with food, the choice of chemical blowing agents is limited. The most commonly used chemical agents in this case are various food ingredients, like fatty acids, vegetable fats, fatty esters, talc, and sodium bicarbonate. Sodium bicarbonate decomposes at a low temperature (145–150 °C) and carbon dioxide is released [14], while its combination with the fatty acids and esters having melting points from 50 to 200 °C is expected to influence the gas evolution.

However, introduction of sodium bicarbonate and organic acids in powder form find limitations in injection molding of polypropylene, due to inability to produce the proper master batches since their decomposition process is completed at the PP processing temperature. As an alternative approach coating of PP pellets was attempted using a water-based system using polyethylenoxide (PEO) as the supporting agent. Furthermore, coating of PP pellets was performed using lipid systems.

Another challenge for producing PP foams by injection molding is the type of technology used. Whereas new injection molding technologies have been introduced for the production of polymer foams [15,16,17], the use of standard injection molding technology without any modification can be a challenge for the production of such material [15]. Making use of modified injection molding machines and chemical blowing agents, which cannot be used to produce material intended for contact with food, it has been possible to produce foams with high void fractions [17,18,19,20]. Void fractions in this case reach even 60% [18]. Such blowing agents include azodicarbonamide which has been prohibited for use in foods and in packaging materials intended to come into contact with food in many countries including the EU since 2005 [21]. Using world-wide authorized food grade chemical blowing agents and modified injection molding methods, it has also been possible to reach significant void fractions for relatively thick parts (36% for material thicknesses of 2.6 mm) [22]. Nevertheless, to the best of the authors’ knowledge, it is not known whether it would be possible to produce PP foams using typical injection molding machines and food grade ingredients and decrease significantly PP mass or confer thermal insulation properties to the package.

In the present work, polypropylene (PP) was combined with food grade foaming agents like sodium bicarbonate and fatty acids and esters. For that combination, two coating methodologies were followed using either polyethylenoxide (PEO) or fatty esters as the binding factors for the PP pellets coating stabilization. Furthermore, PP compositions with certain amounts of sodium bicarbonate and different fatty acids or esters were used for the production of the foamed PP based products. Initially a laboratory batch injection machine was used and material with selected compositions, presenting a promising behavior, in terms of pore formation, were transferred to industrial injection processing. The obtained articles and products were characterized in respect to the porous formation using microscopic techniques like SEM and CLSM, and composition/thermal characterization using ATR-FTIR and TGA. Finally, their thermal insulation properties were tested and the void fraction created in the polymer matrix was determined.

## 2. Materials and Methods

### 2.1. Materials

Polypropylene (412 MK49 by SABIK, Riyadh, Saudi Arabia) with a melt flow rate of 45 g/10 min (determined at 230 °C/2.16 kg by SABIK) was used for the experiments. The acids (citric acid—purity 99% and stearic acid-purity 95%), as well as polyethylene oxide (PEO) with MW from 200 k to 4 M and talc were purchased from Sigma-Aldrich (Munich, Germany) and used as received. Palm oil and emulsifiers were purchased from Soya Hellas S.A. (Athens, Greece). Food grade NaHCO_3_ was purchased from the local food market (PENTE S.A., Athens, Greece). Ultra-pure water was obtained by means of an SG apparatus water purification unit.

### 2.2. Preparation of Modified Materials

#### Coating of Polypropylene Pellets

A. Coating of PP with NaHCO_3_ using polymeric binders

A1. PP/NaHCO_3_ 10%/PEO 2%

Air was blowing in a cylindrical vessel of 5 L containing 300 g PP pellets dispersed in a solution of 30 g NaHCO_3_ and 6 g of PEO (MW-1 M) in 300 mL distilled water. The vessel was rotating on an apparatus with two oppositely rotating cylinders. The mixture was rotated under continuous air flow at room temperature. The coated pellets were further dried in an oven under vacuum at 40 °C for 1 day.

A2. PP/NaHCO3 (10%)/PEO (x%)/stearic acid (10%)

The same experimental procedure as above was followed. Stearic acid was dispersed in the NaHCO_3_ and PEO solution. The final dispersion remained under stirring and then added to a vessel containing 100 g PP.

B. Coating of PP with NaHCO3 using lipids as binders

Α round bottom flask containing 180 g of PP pellets was heated in an oven at 80 °C for 1 h while a mixture of fatty esters 7.5 g of palm oil and 7.5 g of food grade emulsifiers mono- and diacylglycerols of fatty acids (E471) and acetic acid esters of mono- and diglycerides of fatty acids (E472a) was melted at 80 °C. 5 g of NaHCO3 was powdered using a ball-mill. Then n-heptane was added to the fatty ester mixture (at a 50% *w*/*w* concentration with respect to the fatty ester mixture) and the powdered NaHCO3 (at a 33.3% *w*/*w* concentration with respect to fatty esters) was dispersed in the fatty ester/h-heptane mixture. The NaHCO_3_ lipid phase dispersion was added to the PP pellets and n-heptane was evaporated using a rotary evaporator (BUCHI, Flawil, Switzerland, Rotavapor R-200) at 70 °C and 25 mbar for 30 min. The final concentration of NaHCO3 in the coated PP pellets was 2.5%.

C. Coating of PP with organic acids using lipids as binders

Three types of systems containing organic acids with lipids as binders were developed: (a) PP/stearic acid systems (5% *w*/*w* concentration in stearic acid), (b) PP/citric acid/fatty ester systems (4% *w*/*w* concentration in citric acid, 2.5% *w*/*w* concentration in palm oil, 2.5% *w*/*w* concentration in E471 and E472α), (c) PP/citric acid/stearic acid systems (2% *w*/*w* concentration in citric acid and 5% concentration in stearic acid).

In the case of the PP/stearic acid system, stearic acid was first diluted with heptane at a 2/1 stearic acid/heptane *w*/*w* concentration and then mixed with the PP pellets. In the case of the systems containing citric acid, citric acid was first ground to powder, then added to the lipid/heptane system as described above and then the PP pellets were added. The coating was formed under heptane evaporation and cooling at room temperature with a rotary evaporator following the same procedure as for the PP/NaHCO_3_/lipid systems.

### 2.3. Preparation of Foamed Materials (Injection Molding)

#### 2.3.1. Preparation of Foamed Materials in Lab-Scale

Polymer foamed samples were prepared in a laboratory injection molding machine (EMCO Injection Molder Model 333, Educational Machinery Corp. Pelham, NY, USA). The mixture with the polymer (pellets or powder) and foaming agents were added into the barrel and heated to reach the desired temperature for 1–2 min. Then the system remained at that temperature for 1–3 min and injected to mold that was kept at room temperature. The barrel temperature was set at either 220 or 240 °C. Optimum conditions were preheating for 1 min and 2 more minutes at 240 °C before injection.

#### 2.3.2. Preparation of Foamed Materials in Large-Scale

Foamed samples in large scale were produced using Stork IMM 1500–850 (Stork IMM, Hengelo, The Netherlands, clamping force: 1500 kN, injection rate: 1590 cm^3^/s, ejector type: hydraulic) and 2500–1450 injection molding machines (Stork IMM, Hengelo, The Netherlands, clamping force: 2500 kN, injection rate: 2124 cm^3^/s, ejector type: hydraulic). Caps (height: 12 mm and thickness: 0.5 mm) and bowls (external diameter: 94.7 mm, height: 58.8 mm—thickness: 0.5 mm and external diameter: 180.9 mm, height: 75 mm—thickness: 0.6 mm). Table 1 presents the processing conditions used in the experiments.

### 2.4. Characterization Techniques

#### 2.4.1. Thermal Analysis

Thermogravimetric analysis (TGA) was carried out in alumina crucibles in a Labsys™TG (Caluire, France) apparatus of Setaram from 25 to 800 °C under nitrogen and at a heating rate of 10 °C/min.

#### 2.4.2. Attenuated Total Reflection Fourier Transform Infrared Spectroscopy (ATR-FTIR)

The ATR-FTIR spectra of the mixtures were recorded using Bruker Optics’ Alpha-P Diamond ATR Spectrometer of Bruker Optics GmbH (Ettlingen, Germany) and Thermo Nicolet 380 IR spectrometer operating with a SmartOrbit reflectionaccessory (Thermo Electron orporation, Madison, WI, USA).

#### 2.4.3. Scanning Electron Microscopy (SEM)

Scanning electron microscopy of polymer foams (SEM, JEOL 6300, Tokyo, Japan) was performed to investigate their porous structure. The cryofractured surfaces were sputtered with gold to produce electric conductivity before SEM examination.

#### 2.4.4. Confocal Laser Scanning Microscopy (CLSM)

The porous structure of materials developed both in the laboratory and on a large scale was examined using confocal laser scanning microscopy. The Carl Zeiss model LSM 700 confocal laser scanning microscope (Carl Zeiss Microscopy GmbH, Jena, Germany) was used to perform the measurements. Specifically, the sample was mounted on a glass slide vertically using a blue-tac adhesive in order to observe the cross-sectional area. The test was performed using the 405 nm laser in reflectance mode without the use of filters and without the need for staining the samples, in order to be able to display the relief of the section surface. The objective lenses used were 5×, 10×, 20× and a digital zoom of the scanned area was also used.

#### 2.4.5. Heat Transfer Parameters

In order to evaluate heat transfer through the large-scale samples side walls the following procedure was used: 250 g of water was heated at 80 ± 1 °C and the examined containers (PP control sample and PP foamed samples) were in environment temperature. A self-adhesive thermocouple (Type K, Molded Silicone Design, AWG 24 (0.511 mm), Omega) was attached on the outside wall of the studied samples at middle height of the specimen and then the heated water was added. The thermocouple was connected to an RD9912 system (OMEGA Norwalk, CT, USA) interfaced to a PC. On-line temperature measurements were acquired at 2 Hz.

#### 2.4.6. Thermal Conductivity

The thermal conductivity of the materials developed in large scale was measured using the apparatus presented in Figure S1. The apparatus was constructed according the ASTM C 518–98 method: “Standard Test Method for Steady-State Thermal Transmission Properties by Means of the Heat Flow Meter Apparatus”. In brief, the apparatus consisted of two Al plate assemblies (5 cm × 5 cm). A heat flux transducer (HFS-3, thickness: 0.18 mm, Omega) and a thermocouple (Type K, Teflon insulated, AWG 30, 0.25 mm, Omega, Norwalk, CT, USA) were flush mounted on the inner surface of each plate. During the measurement a sample of the same size (5 cm × 5 cm) was placed between the plates and came into contact with the heat flux transducer and thermocouple. The lower plate (“hot plate”) was placed on a thermoregulated heat source. The heat flux transducers and the thermocouples were connected to an RD9912 system (OMEGA, Norwalk, CT, USA) and further interfaced to a PC. On-line heat flux transducer output and temperature were acquired at 2 Hz. Calculations of the thermal conductivity were performed using the equations provided in the ASTM C 518–98 method with values at steady state conditions (i.e., temperature variation ±0.0 °C in both plates and heat flux sensor output variation ±0.05% for both sensors).

## 3. Results

### 3.1. Coating with Individual Ingredients

Coating of PP pellets with sodium bicarbonate was attempted using different coating methodologies. These methodologies used either PEO or food grade lipids carriers of NaHCO_3_ to form the coating. For the PEO approach the following coating methodology was used. Polymers in pellet form like polypropylene pellets were combined with aqueous solutions of NaHCO_3_ and polyethylene oxide (PEO) that was used in order to improve the adhesion of NaHCO_3_ to the polymer pellets and stabilize the coating. For the improvement of uniformity of the coating on the pellets different molecular weights (*M*_W_ = 200,000, 300,000, 600,000, 1,000,000 and 4,000,000) of the polyethylene oxide were studied. The best coating was obtained when the water soluble polymer (PEO) had molecular weight *M*_W_ = 1,000,000. Initial experiments were performed in a rotary evaporator and in second stage a cylindrical vessel equipped with air inlet and outlet was used that was rotating on a mill rotating system at room temperature. Finally, the resulted coated pellets were dried at 40 °C.

For the lipid film approach, the most effective approach for the formation of lipid coating was the direct dispersion of finely ground NaHCO_3_ in a palm oil/n-heptane mixture. Heptane was subsequently evaporated under vacuum with simultaneous rotational mixing, then lipid partial crystallization took place to temperature decrease from 70 °C to room temperature to form the final coating. The addition of n-heptane aided to decrease the lipid phase viscosity. This was important in order to homogeneously disperse NaHCO_3_ at temperatures much lower than its decomposition and chiefly to form a more uniform and thin film during partial lipid crystallization along the evaporation-coating process. Of course, n-heptane could be substituted with hexane or another economic organic solvent in an industrial process but n-heptane was chosen herein for the safety of personnel and for a better control of the sample preparation steps due to its relatively low volatility at room temperatures.

### 3.2. Combination of Sodium Bicarbonate with Fatty Acids

**Figure 1:**
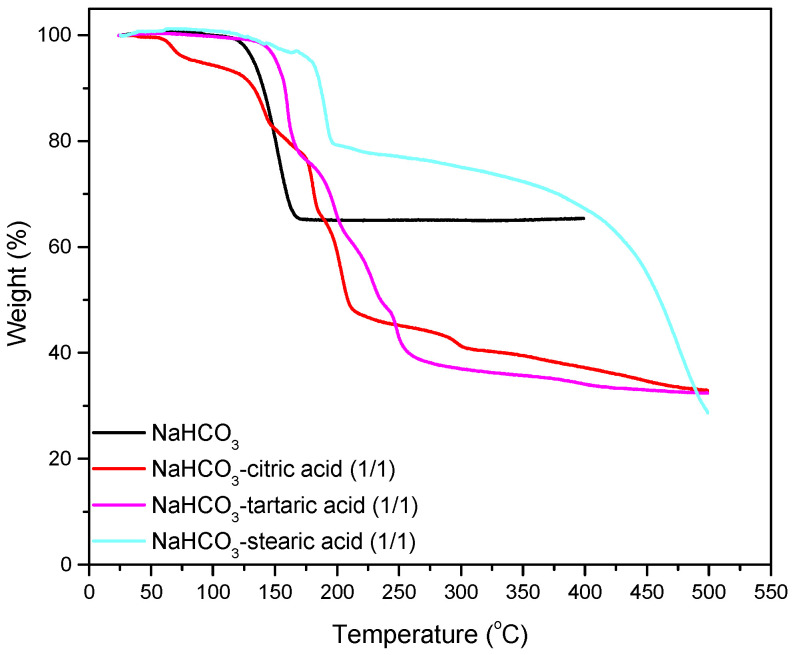
Thermogravimetric analysis of NaHCO_3_ (black line) and mixtures NaHCO_3_/citric acid (1/1) (red line), NaHCO_3_/tartaric acid (1/1) (magenta line) and NaHCO_3_/stearic acid (1/1) (green line).

In order to enhance the gas formation from the sodium bicarbonate decomposition, its combination with fatty acids and other food-grade organic acids was examined. Different additives combinations were tested in various weight ratios. Representative results are shown in Figure 1 and Table 2.

SB: NaHCO_3_ → Na_2_CO_3_ + H_2_O + CO_2_

CA: C_6_H_8_O_7_ → C_6_H_6_O_6_ + H_2_O

Mixture SB/CA: C_6_H_8_O_7_ + 3NaHCO_3_ → C_6_H_8_Na_3_O_7_ 2H_2_O + 3CO_2_ + H_2_O

Additives such as organic acids, are food ingredients and were used for the best foaming of NaHCO_3_ (SB). For CO_2_ releasing systems the citric acid (CA) is in many cases an endothermic, water soluble acid of choice. The mixture NaHCO_3_/citric acid (1/1) has been previously studied [10]. Decomposition of both compounds and reaction between them is expected to take place in the range of temperatures used during PP processing. Reactions, like the one shown below, lead to carbon dioxide and water vapors generation leading to a 50% weight loss of its original mass after thermal decomposition.

On the other hand, the NaHCO_3_/stearic acid system decomposed in a narrow temperature range but maintained 80% of its original mass (green line) as opposed to the other two systems that retain approximately 40% of their original mass. Matching of the decomposition characteristic of these systems with the polypropylene process temperature and other process conditions like time and pressure have to be considered for the application to large-scale systems.

### 3.3. Coating with Combined Ingredients

Based on the above results, systems containing combined ingredients were developed both using PEO and lipids as carriers in different systems. The PEO based approach for the combined coating of PP pellets with an organic acid along with NaHCO_3_ was used. In this case, citric acid was rejected because it is water-soluble, so it can react with NaHCO_3_ during the coating process. Thus, stearic acid was chosen, since this remains insoluble in the water and can be incorporated in the coating without reacting with sodium bicarbonate.

The lipid-based approach consisted of replacing palm oil with stearic acid in order to form a stearic acid/NaHCO_3_ coating. Since an aqueous phase was not used in this approach systems containing NaHCO_3_ and citric acid or both citric acid and stearic acid were developed using the procedure described above.

### 3.4. Examination of the Foaming Compositions in Laboratory Scale

Having the above results in mind, the systems NaHCO_3_/citric acid, NaHCO_3_/stearic acid, NaHCO_3_/stearic acid/citric acid coated individually on PP pellets as well as their combination with fatty esters were chosen since their decomposition is completed in temperatures lower that the polypropylene processing temperature. The two selected acids differ on their melting points from 70 °C for stearic to 153 °C for citric acid their 1 to 1 ratio to the sodium bicarbonate gives high degree of gases formation up to 50 wt% as shown in Table 2. The introduction of fatty esters is expected to influence the PP melt flow and gases evolution and subsequently the pore formation.

**Figure 2:**
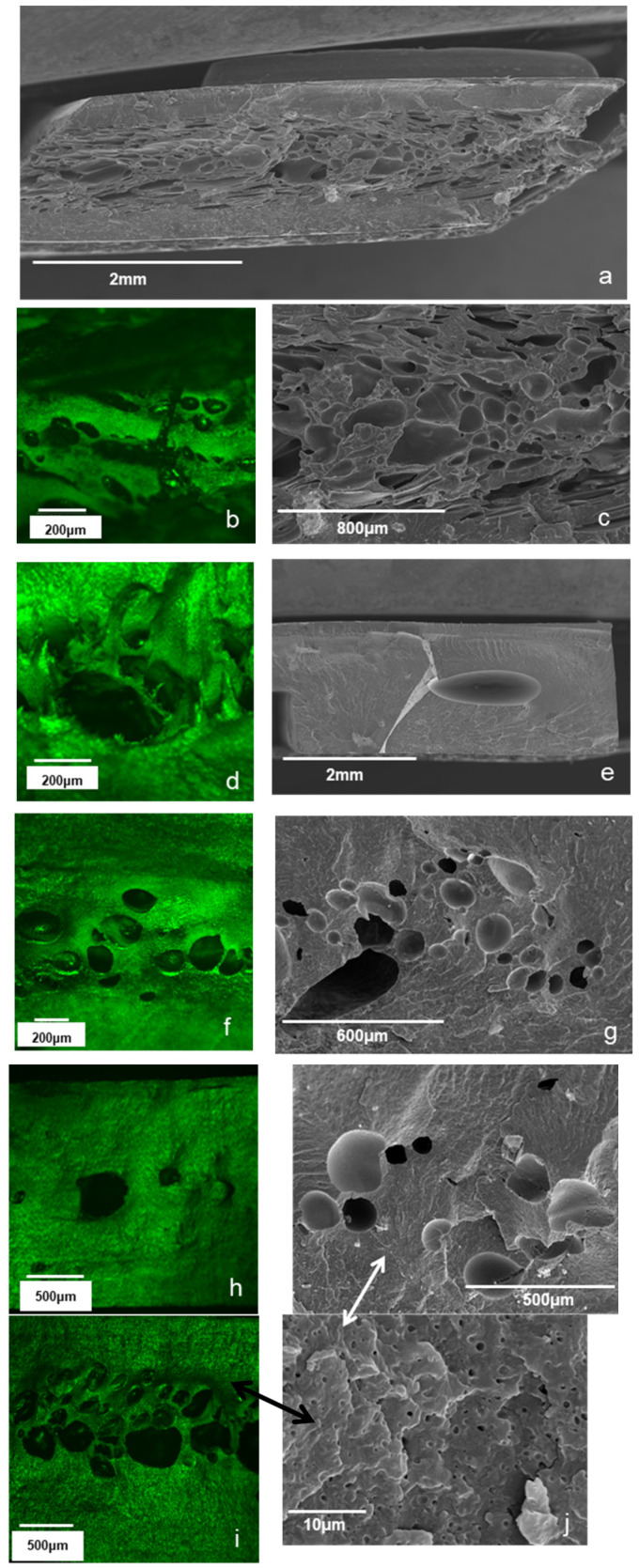
SEM (**a**,**c**) and CLSM (**b**) images of Sample S1, CLSM (**d**) and SEM images (**e**) of Sample S2, CLSM (**f**) and SEM images (**g**) of Sample S4, CLSM (**h**) image of sample S5, CLSM (**i**) and SEM images (**j**) of Sample S6.

Initial experiments, in order to examine the effectiveness of the coatings, were performed in a laboratory injection machine. The different compositions of PP pellets individually or combined coated with the certain amounts of sodium bicarbonate and acids were placed into the heated barrel at a standard temperature of 240 °C. These blends were melted for different time intervals from 1 to 3 min and then injected into a mold under pressure. The different studied systems are gathered in Table 3. More specifically, the concentration of NaHCO_3_ varied from 1.3 to 2% *w*/*w*, the citric acid concentration from 1 to 2% *w*/*w* and the stearic acid content from 0.5. to 1% *w*/*w*. In the case were acid blends were used the total acid concentration varied from 0.5 to 2% *w*/*w* at a constant citric/stearic acid weight ratio of 2/5. The resulting objects were examined using microscopic analysis (SEM and CLSM) in order to evaluate the porous structure formation. Different retention times were tested in order to find the optimum conditions for the formation of the foamed samples. Figure 2 presents microscopy results on the porous structure of selected samples with pores ranging from approximately 1 μm to a few hundred micrometers.

The best results were obtained for S1, where both acids were used in combination with NaHCO_3_, both at a concentration of 2% (Figure 2a–c). Reducing the concentration of acids in the blend resulted in an increased time for foaming and the formation of larger pores at lower void fractions without a homogeneous pore structure (S2. Figure 2d,e). Further reducing the acid concentration resulted in the formation of no voids at all under the same experimental conditions with S1 and S2 (not shown). The effect of the acid type on foam formation can be observed by comparing the results of Sample S4 to S5 (Figure 2e,f, respectively). The addition of citric acid induced foaming earlier during processing. Furthermore, a more homogeneous pore structure and higher void fractions was obtained by citric acid addition, the latter being in line with the results of TGA (Figure 1, Table 2). Furthermore, the overall composition affected the time when the onset of foaming was observed in the samples: samples with a higher concentration in fatty acids and fatty esters (Samples S4, S5, S7) presented lower foam formation times. This was probably due to the effect of fatty esters and fatty acids on the onset of melting and on the melt viscosity which brought NaHCO3 and the acids earlier into contact with one another.

Based on that preliminary examination, compositions that showed foaming in the laboratory batch extruder were prepared in 1–2 kg scale in order to be examined in an industrial scale. The results for these systems are presented in Section 3.3.

### 3.5. Examination of the Foaming Compositions in Industrial Scale

#### 3.5.1. Effect of Scale-up and First Screening of Process Variables on Pore Formation

The most successive compositions according to the results of small-scale experiments were examined in large scale. Changing the scale in the experiments induces a significant change in the variables under which the additives decompose and/or react. Namely, the time–temperature profile is significantly different, with process time being approximately ten times lower compared to the small-scale experiments. Furthermore, mixing conditions are different with more intense mixing and high shear stresses in the large-scale experiments. Finally, a very important variable that is expected to affect not only the reactions but also bubble retention and distribution in the low viscosity polymer melt is pressure.

The first consequence of the scale-up (and the consequent change in the process variables) was the significant reduction in the void fraction or the absence of pore formation, even for samples that, in the laboratory scale experiments, showed high void fractions. Therefore, it was considered significant to examine the effect of process variables on the characteristics of the polymer foam. The process variables examined were the injection speed and pressure, the screw rotation speed, the temperature of the mold and oven as well as the residence time and temperature in the mold. These first experiments were performed according to the variables presented in Table 4.

**Figure 3:**
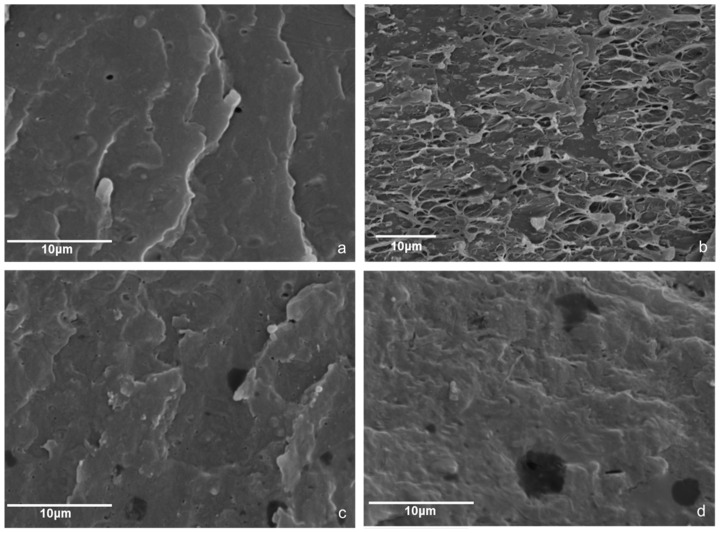
Scanning Electron Microscopy (SEM) images of Cryofractured Sample containing 2% NaHCO_3_, 1.4% stearic acid and 0.6% citric acid produced under different temperatures (namely (**a**) at 220 °C and (**b**–**d**) 240 °C) and under different injection speeds (namely (**a**) and (**b**) at 300 mm/s, (**c**) at 200 mm/s and (**d**) at 100 mm/s.

**Figure 4:**
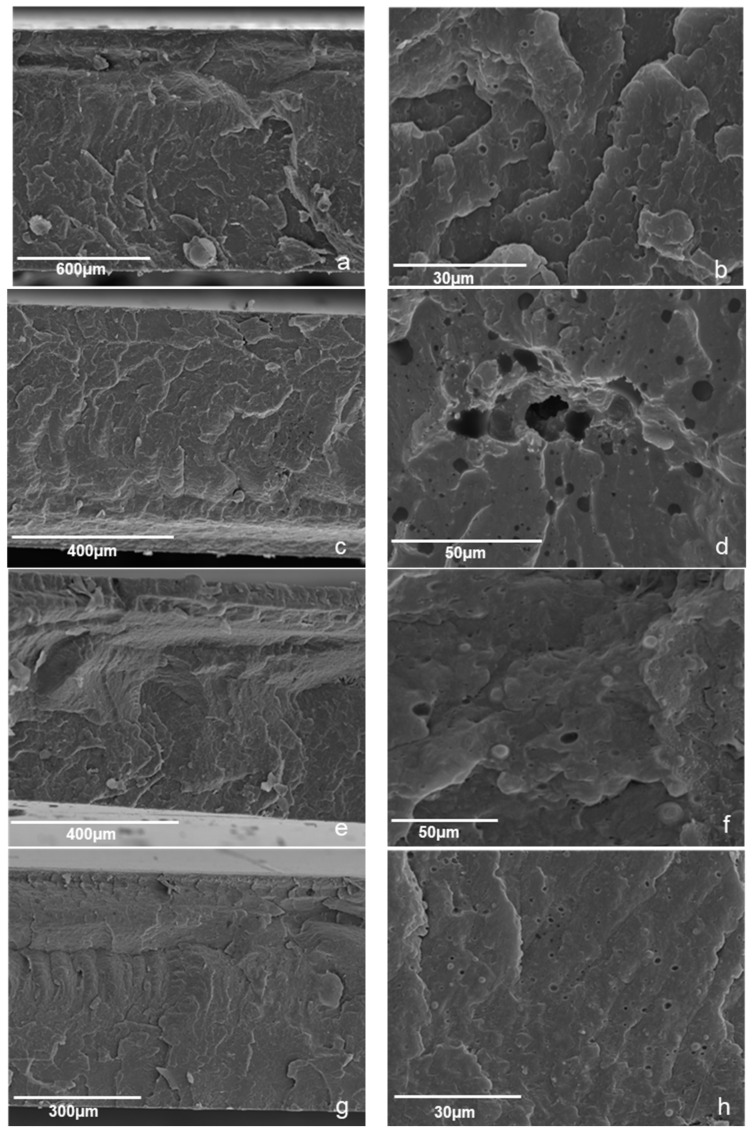
Scanning Electron Microscopy (SEM) images: (**a**,**b**) control PP, (**c**,**d**) sample S4 (injection speed 500 mm/s, injection pressure 100 bar), (**e**,**f**) sample S1 (injection speed 100 mm/s, injection pressure 150 bar) and (**g**,**h**) sample S1 (injection speed 300 mm/s, injection pressure 150 bar).

Figure 3 presents indicative results on the effect of process variables on the microstructure characteristics of the foams. It is observed that increasing the process (oven) temperature from 220 to 240 °C induced an increase in bubble formation (compare Figure 3a,b) for the same material composition. Figure 3b–d presents the effect of injection speed on indicative samples. From the samples presented in Figure 3, as well as from the rest of the samples, it was observed that the injection speed was a significant process variable, which had an effect on the bubble size, void fraction and distribution within the polymer mass, whereas the effect (e.g., increase or decrease in bubble sizes) and the intensity of the effect depended on the rest of variables. It should be noted that, within the range of process variables used, no changes in the microstructure of control PP were observed. As can be observed in Table 4, both bowls and caps were formed under varying conditions. Figure 4 presents results on the samples produced using PP coated with NaHCO_3_ and organic acids as additives. The void fractions of the bowls and caps were estimated by measuring the weight ratio of the samples vs. a control void fractions for the caps ranged from, 1 to 10% and for the bowls up to 5%. The reasons for the differences between caps and bowls lay not only on the differences in the process variables (rotation speed, pressure etc.) that had to be used in order to form the different parts but probably also on the different thickness of the parts allowing more space for the foam to develop in the thicker caps. Among the process variables that were used for the production of different samples in this set of experiments, the ones that showed a significant effect were the injection speed, the screw rotation speed and the oven and mold temperature.

The resulting samples were tested using Attenuated Total Reflection Fourier Transform Infrared Spectroscopy (ATR-FTIR) and Thermogravimetric analysis (TGA) in order to check if the sodium carbonate had decomposed or not during the production process. The samples were also tested for their mechanical properties by tensile tests and for their morphology was studied by Scanning Electron Microscope (SEM) after fracture in liquid nitrogen.

**Figure 5:**
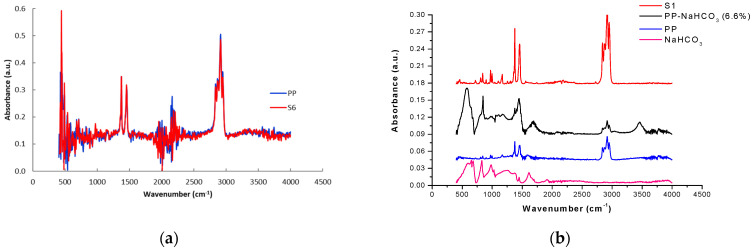
FTIRATR spectra of (**a**) pure PP package and sample S6 and (**b**) NaHCO_3_, PP, PP/PEO/NaHCO_3_ and sample S1.

**Figure 6:**
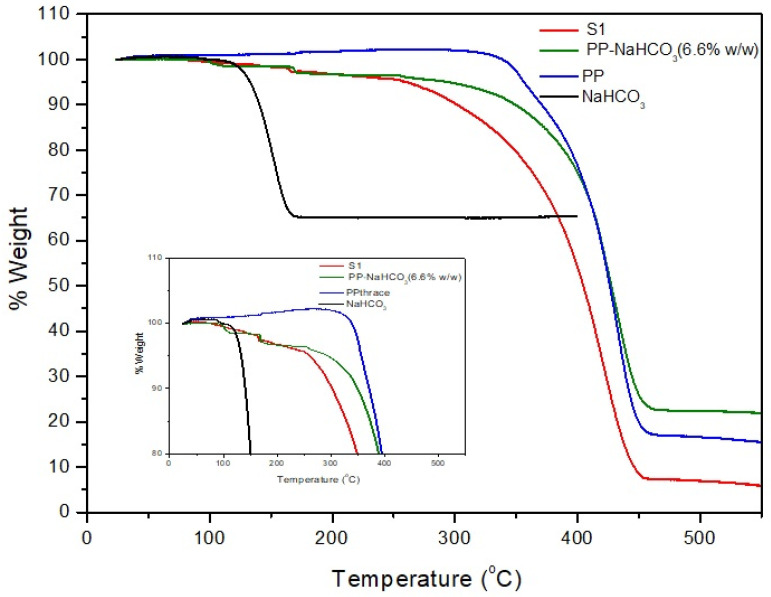
Thermogravimetric analysis (TGA) of NaHCO_3_ (black line), mixture PP/NaHCO_3_ (6.6% *w*/*w*) (green line), pure PP (blue line) and Sample S1 (red line). Insert picture: magnification of the same data for temperatures 80 to 110 °C.

Results of ATR-FTIR showed no difference between the surface composition of the pure PP packaging and of the one with NaHCO_3_ and organic acids for both PEO coated and lipid coated systems (Figure 5). Due to the fact that the ATR-FTIR analysis focuses on the surface of the sample, the presence of deposited NaHCO_3_ on the raw materials is confirmed, as it can be seen but the amount of unreacted NaHCO_3_ in the final products cannot be detected through this technique. For that reason, the samples were studied through thermogravimetric analysis (TGA). The results showed that, in several cases, the NaHCO_3_ remains unreacted probably due to the very short time that the process takes place (Figure 6).

#### 3.5.2. Effect of Composition and Fine Tuning of Industrial Scale Process Variables on Pore Formation and Thermal Properties

The experiments and measurements presented in Section 2.3.1 helped at defining optimum conditions for injection molding in order to form pores within the PP matrix. Given these variables, new experiments were performed where a change in the composition as well as an extensive change in the range of variables (refer to Table 1) was applied in order to increase the void fraction within the PP matrix. Critical properties like void formation and insulation ability were tested thereafter.

The interest in formation of bubbles and voids in the mass of polymeric packaging material can prove advantageous since bubbles can confer (a) a decrease in the use of polymeric material mass and (b) an increase the insulation properties of packaging materials. To this end, for samples with the best bubble formation according to the microscopy techniques, the void fractions and heat transfer parameters were also determined.

**Figure 7:**
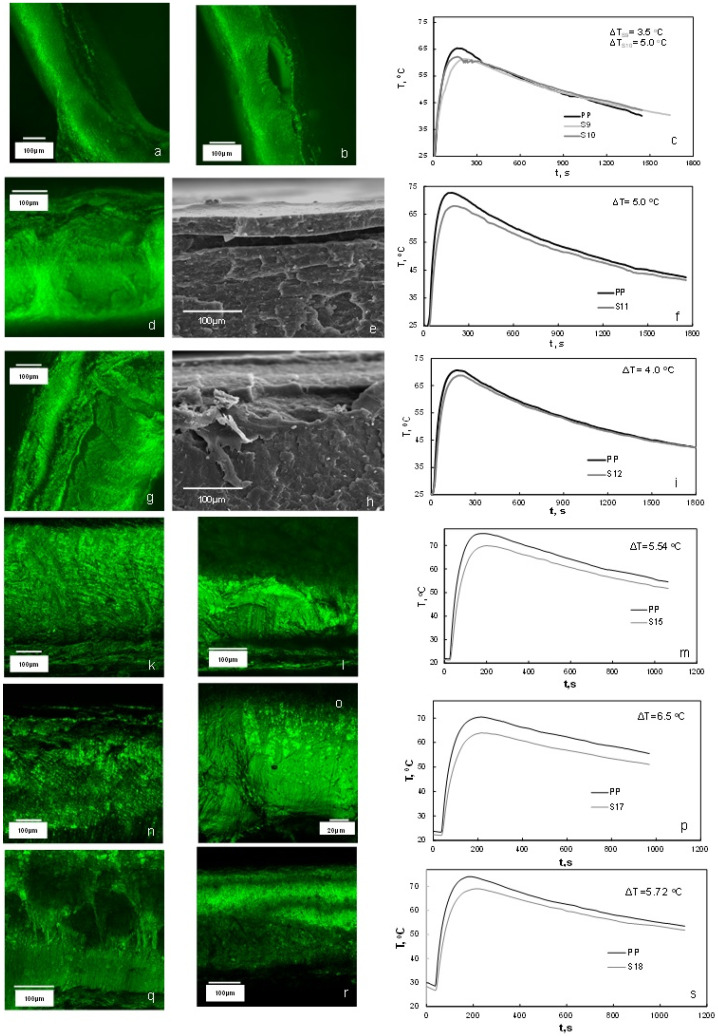
CLSM (color) and SEM (greyscale) images of samples taken at the side wall of samples S9 (**a**) S10 (**b**) S11 (**d**,**e**), S12 (**g**,**h**) and at the bottom and wall of samples S15 ((**k**)-bottom, (**l**)-wall), S17 ((**n**)-bottom, (**o**)-wall) and B101b ((**q**)-bottom, (**r**)-wall). Next to the microscopy images, the corresponding temperature profiles are presented: (**c**) for S9, S10 (**f**) S11, (**i**) S12, (**m**) S15, (**p**) S17 and (**s**) S18. For the compositions of samples and process variables refer to Table 5.

**Figure 8:**
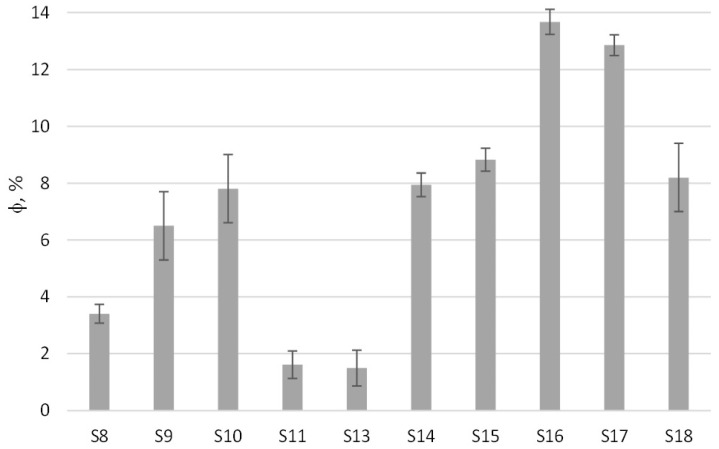
Void fraction (Φ) of samples S8, S9, S10, S11, S13, S14, S15, S16, S17 and S18. For the compositions of samples and process variables refer to Table 5.

**Figure 9:**
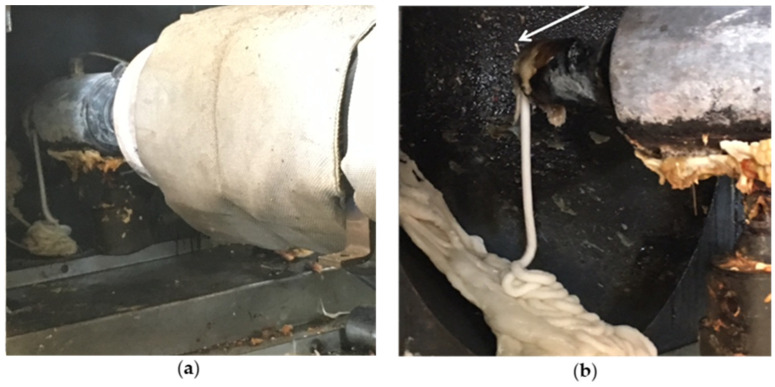
Extrudate in the presence (**a**) and absence (**b**) of foaming additives.

Figure 7 presents SEM and CLSM results as well as the time-temperature profiles obtained at the wall of the cups after filling them with hot water. The compositions and processing conditions used for producing these samples are presented in Table 5. The temperature differences obtained when hot water was poured in a control package compared to the foamed one for our experiments reached 6.5 °C. Figure 8 presents the void fractions obtained in the foamed bowls, which were calculated using weight measurements of control samples and foamed ones. Herein, foam formation was represented in terms of void fraction (instead of density or specific gravity) because the new packages included in many cases materials that do not decompose and have significant differences in density. The void fractions obtained for the samples developed in this work reached 14%, which can be considered satisfactory given the type of material, process and package thickness. The above packages presented lower thermal conductivities with respect to the pure PP sample (Table 6). The observed decrease in thermal conductivity reached 20% for Sample 17b. For the same sample, the thermal conductivity (Table 6) was close to this of thermal insulation material (<0.1 W/m·K [23]). Comparing the results of the thermal conductivity with the ones of void fraction, it can be understood that thermal insulation in the packages was not conferred only by the void fraction. Samples S17 and S18, which presented the lowest thermal conductivities, were produced in addition of talc which seems to have affected the thermal conductivity. Regarding void formation, it was observed from the SEM/CLSM images (but also macroscopically) that the bottom of the cup had the larger pores and higher void fractions. A reason for this could be the differences in shear during the filling of the bowls, which can be expected to affect bubble coalescence and their removal out of the package mass. Such a result can also be related to the relatively low temperature differences (temperatures were measured at the walls) between control samples and foamed ones. In addition, void fractions in the caps were up to 200% higher than the bowls, underlining the significance of process conditions. In the developing foams during injection, molding the injection process had a great effect on the number of pores of the final product since a part of the gas was expelled from the PP mass during injection, as shown from the diameter of the extrudate shown in Figure 9.

In order to reduce the effect of expelling the formed gas from the mold with subsequent reduction of void faction, the piston displacement was limited to a distance so as to allow bubbles within the polymer matrix and produce a fully developed bowl at the same time. The effect of piston displacement can be observed by comparing the void fractions of samples S8 and S9 as well as the ones of S15 and S16 (Figure 8). The cooling time was another significant variable in foam formation, allowing for foam to develop within the mold. The effect of cooling time on the void fraction can be observed by comparing samples S9 and S10. Furthermore, the increase in oven and mold temperature resulted in an increase in void fractions due to the increase in decomposition/reaction rates (compare samples S14 and S15). This was observed also in preliminary large-scale experiments.

Talc was used in some of the samples in order to observe its potential filler and/or nucleating agent effect. Furthermore, talc increases the viscoelasticity of the polymer melt, thus allowing the formed gas to be better entrapped in the polymer matrix [24]. In addition, it aids to the mechanical properties of polymer foams [25]. Among the samples with the highest void fractions were those containing talc. However, similar void fractions were obtained for other reactant compositions without talc. More experiments are required in order to elucidate the effect of talc in our systems.

Apart from the challenges of the process (high pressures, low processing times) and the material (PP has a low melt viscosity), another challenge for increasing the void fraction and the insulating properties of the examined material was the low thickness of the package used for the experiments (0.5, 0.6 mm), which did not allow enough space for bubbles to develop. It was shown previously that the thickness of the material plays a significant role in foam development. Increasing the thickness of the material by 18% resulted in an increase in void fraction of 39% in a core-back injection molding application [22]. Regarding the material properties, changing the structure of PP can lead to a better development and retention of foam [15]. Such an approach could improve the present results.

## 4. Conclusions

This work illustrates the efficient coating of polypropylene pellets with food grade coating agents like sodium bicarbonate and organic acids to create foamed polypropylene articles. Effective coating of PP pellets was achieved through stabilization with PEO or lipids as sodium bicarbonate binders. Different combinations of PP with the foaming agents were tested in a laboratory scale injection molding machine and the most promising compositions were transferred to a large-scale industrial extruder.

Several process variables were found to be critical to the overall success of large-scale extrusion. Specifically, increasing the oven and mold temperature resulted in increased reaction rates and increased foaming. Increasing the cooling time allowed for more time for the foam to develop within the mold. The injection speed and screw rotation speed affected shear and mixing with an interaction between them being observed with respect to their effect on foam/void characteristics.

The displacement of the injection piston (at 100% of the displacement used for pure PP or less) had a significant effect on foam formation, leaving more space for the foam to develop in the mold. Overall, the void fraction achieved for the examined compositions and process reached 14% for the 400 μm specimens of the PP samples containing 4% sodium bicarbonate and 4% citric acid as an example, which was related to the reduction of PP amount used for their production. The new packages presented a decreased thermal conductivity by up to 20%. Finetuning of the process, changing the structure of PP and increasing the thickness of the specimen could result in lower-cost foamed food grade PP articles with improved insulating properties.

## Figures and Tables

**Table 1:** Injection parameters and their values examined during the large scale experiments.

Parameter	Values
Injection speed (mm/s)	100, 200, 285, 300, 320, 500
Screw rotation speed (rpm)	100, 200, 300, 350
Injection pressure (bar)	100, 150
Mold temperature (°C)	220, 230, 240
Oven temperature (°C)	220, 240
Cooling time (s)	1.6, 1.8, 3.6, 8
Piston displacement during injection (mm)	40, 41, 44, 45, 45.8, 46, 48, 52, 53, 54, 55, 56, 70
Machine cycle time (s)	33.7,
Back pressure (bar)	800, 850, 1000, 1252, 1270, 1300

**Table 2:** Decomposition range and weight loss in sodium bicarbonate organic acids systems.

Sample	Decomposition Range (°C)	Weight Loss during Decomposition
NaHCO_3_	110–171	35
NaHCO_3_-citric acid (1/1)	71–214	52
NaHCO_3_-tartaric acid (1/1)	140–263	60
NaHCO_3_-stearic acid (1/1)	139–195	20

**Table 3:** Studied compositions on the Laboratory Injection Machine.

Sample	Quantity of Blend PP/PEO/NaHCO_3_ (6%in NaHCO_3_)	Quantity of PP/veg.fat/NaHCO_3_ (2.5% in NaHCO_3_)	Quantity of Blend PP/Stearic Acid (10%)	Quantity of Blend PP/veg.f Citric Acid (2%)	Quantity of Blend PP/Stearic Acid (5%) and Citric Acid (2%)	Quantity of PP	Time (min)	Macroscopical Observation
S1	1.7 g (2%)				1.4 g (2%)	1.90 g	2:00	Foaming in the molded sample
S2	1.7 g (2%)				0.7 g (1%)	2.6 g	2:00	no foaming
S3	1.7 g (2%)				0.35 g (0.5%)	2.95 g	2:00	no foaming
S4		4 g (2%)		1.3 g (1%)	-	-	1:30	Foaming at 40 s
S5		4 g (2%)	0.5 g (1%)		-	0.5 g	1:20	Foaming at 1:10
S6		4 g (2%)	0.25 g (0.5%)			0.25 g	2:30	Foaming at 2:00
S7		4 g			1.4 g (2%)	-	1:40	Foaming at 1:10

**Table 4:** Conditions used during the first set of large-scale experiments. Sample compositions presented in Table 2 were used for these experiments.

Type of Sample	Screw Rotation Speed (rpm)	Injection Speed (mm/s)	Injection Pressure (bar)
Cap	100, 300	100, 200, 300	150
Cap	200	100, 200, 300, 500	100, 150
Bowl	200, 350	285	150
Bowl	200, 350	200, 285	150
Bowl	200, 350	285	150

**Table 5:** Sample characteristics, compositions and process conditions for the results presented in Figure 7 and Figure 8.

Sample		Reactant Concentrations				Process Conditions					
Code	Characteristics	NaHCO_3_(%)	Stearic Acid (%)	Citric Acid (%)	Talc (%)	Oven Temperature (°C)	Mold Temperature (°C)	Injection Pressure (bar)	Injection Speed (mm/s)	Cooling Time (s)	Injection Piston Displacement (mm)
S8	NaHCO_3_ from PEO coating, stearic & citric acid from lipid coating	3	2.5	1	0	240	240	150	500	1.8	45
S9	NaHCO_3_ from PEO coating, stearic & citric acid from lipid coating	3	2.5	1	0	240	240	150	500	1.8	40
S10	NaHCO_3_ from PEO coating, stearic & citric acid from lipid coating	3	2.5	1	0	240	240	150	500	8	40
S11	NaHCO_3_ and stearic acid from PEO coating	6	10	0	0	240	240	150	500	8	40
S12	Stearic Acid embedded in PP, NaHCO_3_ embedded in LDPE and PP pellets	4	1.2	0	0	240	240	150	500	1.8	45
S13	NaHCO_3_ and stearic acid from lipid coating, citric acid embedded in EVA	2	5	2	0	230	240	150	100	3.6	48
S14	NaHCO_3_ from PEO & lipid coating, stearic acid from lipid coating and embedded in PP, citric acid embedded in EVA, talc embedded in PP	4	5	3	10	220	220	1250	100	3.6	52
S15	NaHCO_3_ from PEO & lipid coating, stearic acid from lipid coating and embedded in PP, citric acid embedded in EVA, talc embedded in PP	4	5	3	10	230	240	1250	100	3.6	52
S16	NaHCO_3_ from PEO & lipid coating, stearic acid from lipid coating and embedded in PP, citric acid embedded in EVA, talc embedded in PP	4	5	3	10	230	240	1250	100	3.6	48
S17	NaHCO_3_ from PEO coating and from lipid coating, citric acid embedded in EVA	4	0	4	0	230	240	1250	100	3.6	45
S18	NaHCO_3_ from PEO coating, stearic acid embedded in PP, citric acid embedded in EVA, talc embedded in PP	4	4	3	8	230	240	1300	100	3.6	46

**Table 6:** Thermal conductivity of the walls of packages. For the compositions of samples and process variables refer to Table 5. The standard deviation of the measurements in all cases was 0.002 W/m·K.

Sample	Thermal Conductivity, k (W/m·K)
PP	0.139
S9	0.122
S10	0.126
S11	0.133
S13	0.116
S16	0.122
S17	0.111
S18	0.117

## Data Availability

Data is contained within the article.

## Supplementary Materials

The following is available online at https://www.mdpi.com/2073-4360/13/2/288/s1, Figure S1: Apparatus for the measurement of thermal conductivity.
