# Supplementary figures and images for: Injection Molded PP Foams Using Food Ingredients for Food Packaging Applications
Source: Polymers (Basel). 2021 Jan 18;13(2):288. doi: 10.3390/polym13020288 (PMC7830478; doi:10.3390/polym13020288)

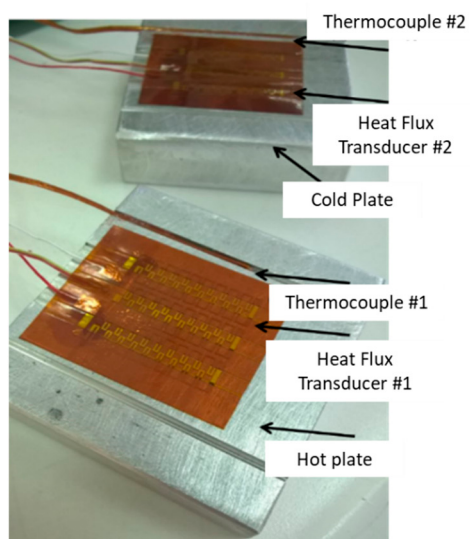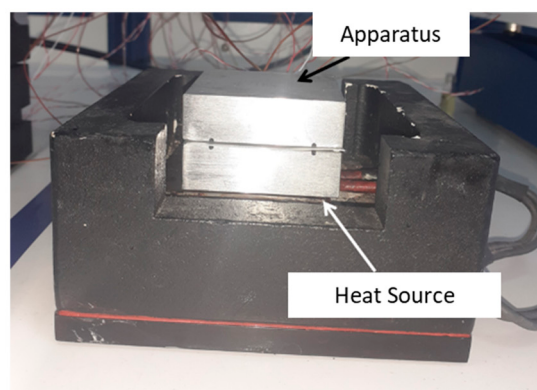

**Figure S1.** Apparatus for the measurement of thermal conductivity

Supplement: Supplementary file 1 [file polymers-13-00288-s001.pdf]
